# Chimeric 14-3-3 proteins for unraveling interactions with intrinsically disordered partners

**DOI:** 10.1038/s41598-017-12214-9

**Published:** 2017-09-20

**Authors:** Nikolai N. Sluchanko, Kristina V. Tugaeva, Sandra J. Greive, Alfred A. Antson

**Affiliations:** 1 A.N. Bach Institute of Biochemistry, Federal Research Center “Fundamentals of Biotechnology” of the Russian Academy of Sciences, 119071 Moscow, Russian Federation; 2 Department of biophysics, School of Biology, Moscow State University, 119991 Moscow, Russian Federation; 3 Department of biochemistry, School of Biology, Moscow State University, 119991 Moscow, Russian Federation; 4 York Structural Biology Laboratory, Department of Chemistry, University of York, York, YO10 5DD United Kingdom

## Abstract

In eukaryotes, several “hub” proteins integrate signals from different interacting partners that bind through intrinsically disordered regions. The 14-3-3 protein hub, which plays wide-ranging roles in cellular processes, has been linked to numerous human disorders and is a promising target for therapeutic intervention. Partner proteins usually bind via insertion of a phosphopeptide into an amphipathic groove of 14-3-3. Structural plasticity in the groove generates promiscuity allowing accommodation of hundreds of different partners. So far, accurate structural information has been derived for only a few 14-3-3 complexes with phosphopeptide-containing proteins and a variety of complexes with short synthetic peptides. To further advance structural studies, here we propose a novel approach based on fusing 14-3-3 proteins with the target partner peptide sequences. Such chimeric proteins are easy to design, express, purify and crystallize. Peptide attachment to the C terminus of 14-3-3 via an optimal linker allows its phosphorylation by protein kinase A during bacterial co-expression and subsequent binding at the amphipathic groove. Crystal structures of 14-3-3 chimeras with three different peptides provide detailed structural information on peptide-14-3-3 interactions. This simple but powerful approach, employing chimeric proteins, can reinvigorate studies of 14-3-3/phosphoprotein assemblies, including those with challenging low-affinity partners, and may facilitate the design of novel biosensors.

## Introduction

The 14-3-3 family of eukaryotic proteins are abundant, medium sized proteins (~30 kDa subunit mass) endowed with a well-characterized phosphopeptide-binding ability^1^. This feature allows members of the family to work in synergy with several protein kinases, which, upon activation, phosphorylate their client proteins to trigger specific recognition by 14-3-3 proteins. This binding event is a key node in many protein-protein interaction networks that regulate a plethora of cellular processes, including apoptosis, cell division, ion channel trafficking, signal transduction, hormonal production and cytoskeleton rearrangements^1–3^. Consequently, 14-3-3 proteins are major players in a range of human disorders, such as cancer and neurodegenerative diseases, making them important targets for drug discovery and therapy.

In all organisms, 14-3-3 proteins are usually present as several isoforms that are encoded by separate genes^1^. The human 14-3-3 family comprises 7 isoforms (β, σ, ζ, γ, τ, ε, η), that form all-helical W-shaped homo- and heterodimers^4–6^. These proteins function as recognition modules that bind a posttranslationally modified segment of partner proteins and, with rare exceptions^7–10^, do not interact with non-phosphorylated partners. More specifically, 14-3-3 s bind protein partners that have phosphorylated serine and/or threonine residues presented in a specific molecular context^11^. Indeed, 14-3-3 proteins were the first phosphoserine-binding modules discovered^12^. Pioneering research using peptide libraries established the consensus motifs I and II, RSX[pS/pT]XP and RXY/FX[pS/pT]XP (X is any amino acid)^13^, respectively, that preferentially interact with 14-3-3. This immediately suggested that protein kinases with overlapping target sequences (e.g., AGC and CAMK family kinases recognizing (R/K)XXS motifs^14^) might co-operate with 14-3-3, regulating its interaction with target proteins. Later discovery of an additional interacting motif III (pS/pTX(X)-COOH), found at the C terminus of several interacting partners, expanded the binding repertoire of 14-3-3 proteins^15^. The on-going research on 14-3-3 partners is constantly expanding the library of binding sequences^16^. For example, it became clear that many 14-3-3 partners do not have Pro/Gly at position +2, differing from the initially defined consensus. Other significantly deviating examples include peptides of p53 (LMFKpT^387^EGPD), histone acetylase-4 (LPLYTSPpS^350^LPNITLGLP) and peptidylarginine deiminase isoform VI (SSFYPpS^446^AEG), for which the structural basis for interaction with 14-3-3 has been derived by crystallography^17–19^.

At present more than 2000 potential 14-3-3 interactors have been postulated^20^, demonstrating involvement of 14-3-3 members in many cellular mechanisms. Computational tools have been developed for prediction of potential 14-3-3 binding sites^20–22^ and calculating binding affinities of each phosphopeptide based on contribution of individual amino acids to the binding stability^16^. The most optimal binding sequence has a positively charged Arg/Lys residue at position −3 from the central phospho-residue while a downstream Gly/Pro at position +2 confers either flexibility or a kink in the peptide conformation necessary for tight interaction in the amphipathic groove (AG) of 14-3-3^13^. Remarkably, usually the equivalent non-phosphorylated sequences fail to bind to 14-3-3, suggesting that affinity is determined predominantly by electrostatic interactions that attract phosphopeptide to the AG during an initial stage of binding^23^. Accordingly, millimolar concentrations of inorganic phosphate or sulfate may significantly inhibit 14-3-3/phosphotarget interactions by competing for binding at the AG^24^.

A significant finding was that 14-3-3 proteins predominantly interact with proteins enriched with intrinsically disordered protein regions^25^ and that the specific phosphorylatable 14-3-3 binding sequences are mostly flexible and disordered. This poses substantial challenges for structural investigation of 14-3-3/partner interactions. Indeed, crystal structures are available for only two complexes of 14-3-3 with relatively complete target proteins, arylalkylamine N-acetyltransferase (PDB ID 1IB1^26^) and the small heat shock protein HSPB6 (PDB ID 5LTW^27^). Limited structural information prevents understanding of the molecular basis for function of this key regulatory node involved in many clinically important signal transduction pathways, decelerating the development of novel therapeutic approaches. For example, such information is vital for finding small molecule modulators of specific 14-3-3/target complexes^28–32^ that won’t affect interactions of 14-3-3 with other targets. Ultimately, it would be important to screen for such modulators of 14-3-3 complexes with a whole diverse range of peptide sequences, including low-affinity peptides mediating transient interactions. In addition, the current lack of structural information prevents delineating a universal “14-3-3 binding law” and understanding molecular details of the selectivity for 14-3-3 interaction with hundreds of competing partners.

Structure determination for the 14-3-3/peptide complexes is often challenged by the low affinity of peptides and/or their limited solubility, preventing formation of complexes with fully occupied binding sites. To aid structure determination, we have developed a streamlined approach based on chimeric 14-3-3 proteins fused to the sequences of interacting peptides. Such chimeric proteins are easy to design and allow rapid production of large quantities of soluble, crystallization quality protein material. Interacting peptide sequences are fused to the C terminus of 14-3-3 through an optimized linker and subsequently phosphorylated during bacterial co-expression with protein kinase A, to yield fully phosphorylated material facilitating binding of a fused phosphopeptide in the AG of 14-3-3. As proof of principle, we produced chimeras for three different phosphopeptides and demonstrated that it is possible to obtain diffraction quality crystals for all of them. This approach provided accurate structural information on 14-3-3/peptide complexes, overcoming the limitations of traditional co-crystallization approaches with synthetic peptides. Importantly, this approach is compatible with high-throughput studies suitable for the wide 14-3-3 interactome. Furthermore, the approach involving chimeric 14-3-3 proteins can accelerate the design of novel biosensors for *in vitro* screening and *in vivo* imaging, as well as construction of extended protein-protein chimeras involving 14-3-3.

## Results

### Design of 14-3-3 chimeras with interacting phosphopeptides

**Figure 1:**
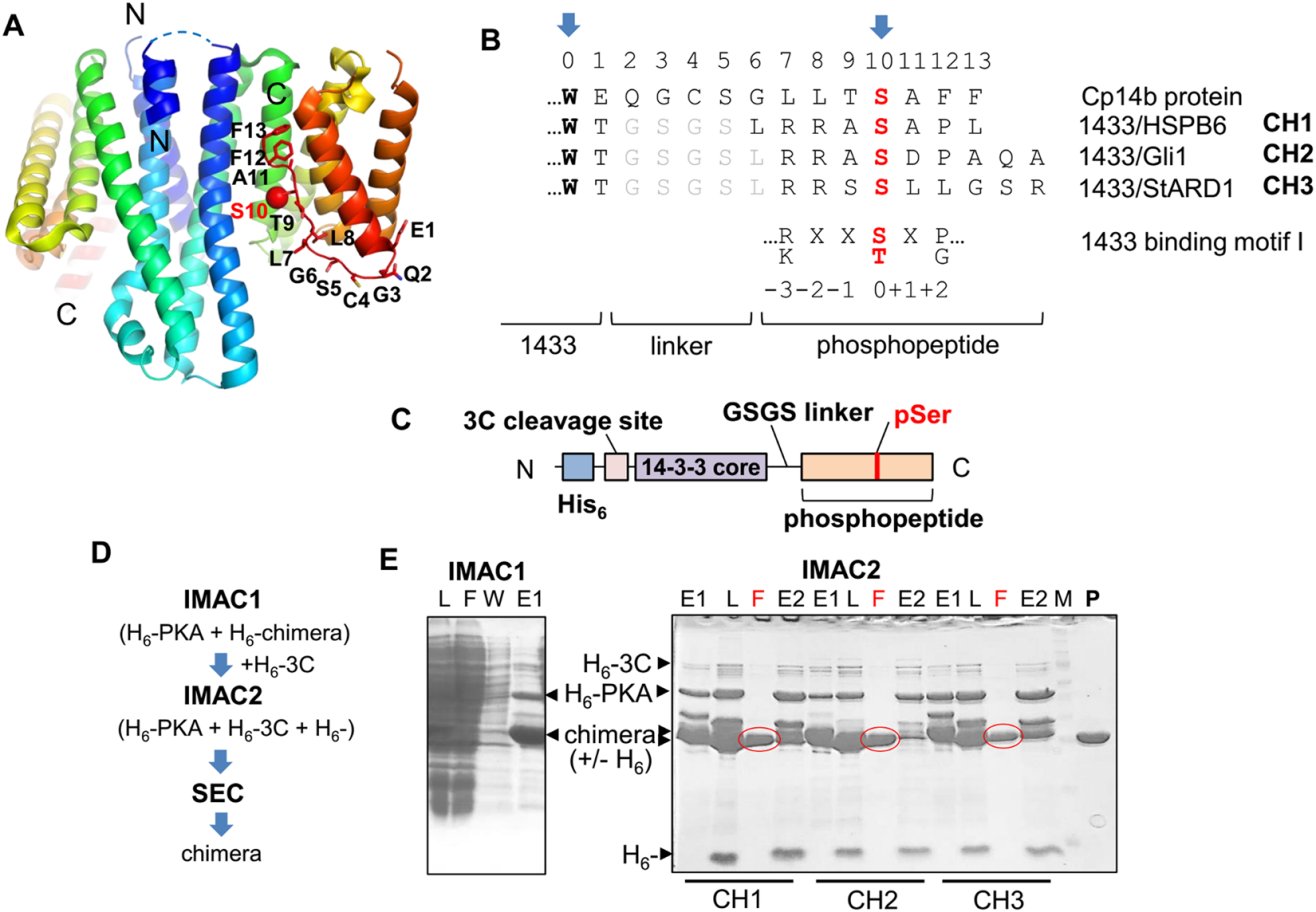
Design and production of the 14-3-3/phosphopeptide chimeras. (**A**) – Crystal structure of the asymmetrical 14-3-3 from *C.parvum* (Cp14b) with phosphorylated flexible C terminal peptide (numbered residues) bound in the AG of one 14-3-3 subunit (PDB ID 3EFZ). Each subunit is colored by gradient from N (blue) to C terminus (red). (**B**) – Alignment of C-terminal regions of Cp14b and chimeras CH1-CH3 showing the linker connecting the conserved Trp (position 0, arrow) of 14-3-3 and the phospho-site (position 10, arrow). Linker sequence is in grey font and the phospho-site is in red font. For comparison, 14-3-3 binding motif I is shown below the alignment. (**C**) – Schematic depiction of the 14-3-3/phosphopeptide chimeras. (**D**) – Purification scheme for obtaining crystallization-ready CH proteins phosphorylated in the course of bacterial co-expression with His-tagged PKA, including subtractive immobilized metal-affinity chromatography (IMAC) for the N-terminal hexahistidine tag removal by 3C protease and size-exclusion chromatography (SEC). (**E**) – Electrophoretic analysis of fractions obtained during IMAC1 and IMAC2 for CH1 (IMAC1) or CH1-CH3 (IMAC2). Lanes are labeled as follows: (L) – loaded fraction, (F) – flowthrough (10 mM imidazole), (W) – wash (10 mM imidazole), E1 – elution at 510 mM imidazole during IMAC1, E2 – elution at 510 mM imidazole during IMAC2. Note the shift of chimera bands as a result of tag removal by 3C (+/− H_6_). Flow through fractions (F) during IMAC2 (red circles) were subjected to additional SEC purification (P – final sample) prior to crystallization.

To probe whether the proposed 14-3-3 chimera proteins fused with different phosphopartner peptides would be amenable for crystallographic studies, we designed a prototypical chimera based on the available crystal structure of the HSPB6/14-3-3σ complex^27^. Thus, the C terminus of 14-3-3σ was fused to the N terminus of the HSPB6 peptide comprising the key Ser16, which is phosphorylated both *in vivo* and *in vitro* by cyclic nucleotide-dependent protein kinases A (PKA) and G (PKG)^33^. An easily crystallizable C-terminally truncated mutant of human 14-3-3σ (Clu3 mutant)^27^ was used as the scaffold for these chimeras. The length of the peptide linker between the 14-3-3 sequence and the phosphopeptide fusion is critical for ensuring interaction. The linker length was informed by structural data on the *Cryptosporidium parvum* 14-3-3, Cp14b protein, where its own C-terminal peptide, phosphorylated during expression in *E. coli*, was bound in one of its AGs (PDB ID 3EFZ)^34^ (Fig. 1A). Despite the uncommon overall fold of this rather exotic 14-3-3 member, it defined a linker of ten residues, between the highly conserved C-terminal tryptophan of 14-3-3 (position 0, Fig. 1B) and the anchored phospho-residue (position 10, Fig. 1B) bound in the AG. The linker used for fusing the HSPB6 phosphopeptide to the C-terminal of 14-3-3σ∆C included: the ordered Thr residue at position 1 (Fig. 1B) that is always present in electron density maps, even for C-terminally truncated 14-3-3 variants; the natural Leu residue preceding the 14-3-3 binding motif of HSPB6 (RRApS^16^APL); and a GSGS segment designed to provide maximal flexibility to create the prototypical 14-3-3/HSPB6 chimera CH1 (Fig. 1B). Additional chimeras of 14-3-3σ∆C were designed to contain peptides from recently described physiological, but structurally uncharacterized, 14-3-3 partners, Gli (chimera CH2) and StARD1 (chimera CH3; Fig. 1B). The three chimeras CH1-3 were expressed as N-terminal His-tag fusions cleavable by the highly specific 3C protease to facilitate their purification (Fig. 1C). To achieve stoichiometric phosphorylation of peptides within the chimeras, we co-expressed them in *E. coli* with the catalytically active subunit of protein kinase A (PKA), known to phosphorylate 14-3-3 binders *in vivo* ^33,35,36^. Importantly, the 14-3-3σ itself, unlike most of other isoforms, is resistant to PKA phosphorylation and subsequent homodimer dissociation^37^, as it does not contain the semi-conservative serine at the subunit interface, which has been reported to destabilize 14-3-3 dimers upon phosphorylation^5,38^.

This design allows large scale co-expression of soluble phosphorylated 14-3-3 chimeras which can be readily purified by standard chromatographic approaches (Fig. 1D). This procedure generated milligram quantities of protein samples that were greater than 98% pure and fully soluble (see Fig. 1E, lane “P”). The properties of the prototypical CH1 chimera were analyzed in some detail, prior to structural studies on all three chimeras.

### Characterization of the 14-3-3/HSPB6 protein-phosphopeptide chimera CH1

CH1 co-expressed with PKA (pCH1) resulted in higher mobility during native gel-electrophoresis than for its unphosphorylated counterpart (Fig. 1A, inset). Likewise, *in vitro* phosphorylation of the latter by PKA resulted in increased electrophoretic mobility, whereas further incubation with alkaline phosphatase partly reversed this effect, suggesting that it is associated with protein phosphorylation and that CH1 can be phosphorylated by PKA both *in vitro* and inside bacterial cells.

**Figure 2:**
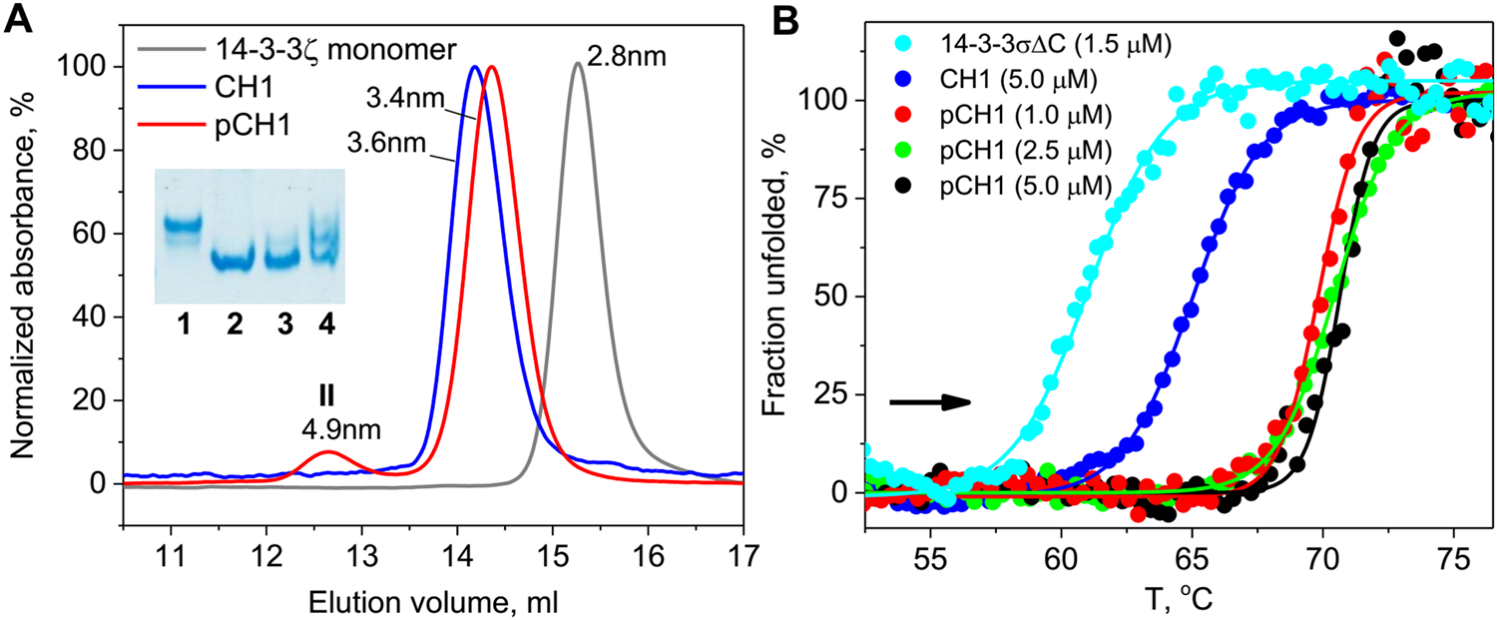
pCH1 characterization. (**A**) – Analytical SEC profiles of the monomeric mutant of 14-3-3ζ and the 14-3-3σ fusion with HSPB6 phosphopeptide expressed in the absence (CH1) or in the presence (pCH1) of PKA, obtained using a calibrated Superdex 200 10/300 Increase column (GE Healthcare). Elution profiles were followed at 280 nm and normalized to absorbance at the peak maxima. Average hydrodynamic radii corresponding to peak maxima obtained from column calibration are indicated. Peaks I and II of the CH1 profile are marked. Inset shows the migration of CH1 (1), CH1 co-expressed (2) or *in vitro* phosphorylated (3) by PKA, or pCH1 *in vitro* dephosphorylated by alkaline phosphatase (4) during native gel-electrophoresis. (**B**) – Heating of 14-3-3σ∆C (1.5 µM), unphosphorylated CH1 (5 µM) or phosphorylated CH1 (1–5 µM) samples from 10 to 80 °C at a constant rate of 1 °C/min followed by intrinsic tryptophan fluorescence (direction is shown by arrow) and analyzed by plotting fraction of unfolded protein versus temperature (See Methods).

The analytical SEC profile for pCH1 contained a major symmetric peak (peak “I”, representing 85–90% of the protein) corresponding to particles with an average hydrodynamic radius *R*_H_ of 3.4 nm and a minor peak (peak “II”) corresponding to particles with the radius of 4.9 nm (Fig. 2A). Comparison with the profiles of a monomeric mutant form of 14-3-3ζ (peak at 2.77 nm consistent with previously reported *R*_H_ value ~2.8 nm^39,40^) and unphosphorylated CH1 (expressed without PKA; single symmetrical peak at 3.6 nm) suggests that peak I of pCH1 corresponds to a dimeric form, whereas peak II corresponds to a higher oligomeric form present in much smaller quantities (10–15%). The apparent smaller radius of the pCH1 dimer (3.4 nm) compared to the 3.6 nm radius of the unphosphorylated protein indicates compaction of the chimera upon phosphopeptide binding. During this transformation the C-terminal lobes of the 14-3-3 core are thought to move relative to the N-terminal base of the protein, to form a closed state upon peptide binding^6,41^. The shift in SEC profile is indicative of formation of this closed or phosphopeptide ‘bound’ state. We can speculate that the small fraction of the larger particles with the 4.9 nm radius is likely due to the concentration dependent cross dimer patching of one chimeric phosphopeptide to another chimeric 14-3-3 dimer to form tetramers (see below).

To further characterize the conformation change between unbound and bound states of the CH1 chimera, we used differential scanning fluorimetry^42^. We compared CH1 chimera with the unphosphorylated control and the core 14-3-3σ∆C dimer (Fig. 2B). In this experiment, unphosphorylated CH1 and 14-3-3σ∆C underwent main thermally-induced transitions with half-transition temperatures of 61 and 65 °C, respectively (Fig. 2B, cyan and blue curves). Under identical conditions, the half-transition temperature for pCH1 was ~70 °C, i.e. 5 or 10° higher, indicating enhanced protein stabilization. This is most likely a result of the phosphopeptide binding into the AG and the resulting overall compaction indicated by SEC. Importantly, lowering the pCH1 concentration to 1 µM did not result in any significant destabilization, indicative of a strong phosphopeptide binding even at low protein concentrations (Fig. 2B). In contrast, addition of equimolar concentrations of untethered phosphopeptide with protein at 1 µM would have resulted in ≤12% of the AG occupancy (given the apparent K_D_ of 6.3 ± 0.5 µM^27^). The apparent increase in binding affinity due to co-localization through fusion with 14-3-3 is highly advantageous for future utilization of 14-3-3 chimeras in biosensor technology, which normally involves low protein concentrations.

### Crystal structure of the prototypical pCH1 chimera

The pCH1 chimera crystallizes under a variety of conditions in several different crystal forms (Table 1). Thus, unlike the natural disordered C-terminal segment of 14-3-3, the phosphopeptide fusion *per se* does not hamper crystallization. One can expect that derivatives of pCH1 with other phosphopeptides will crystallize equally well.

**Table 1:** Crystallization conditions.

Chimera	14-3-3σ Clu3 – HSPB6 phosphopeptide*			14-3-3σ Clu3 – Gli1 phosphopeptide**	14-3-3σ Clu3 – StARD1 phosphopeptide**
Topology	bi-directional peptide swap		self-bound	mono-directional peptide swap	bi-directional peptide swap
Designation	**pCH1℧**		**pCH1X**	**pCH2**	**pCH3**
Crystallization solution (reservoir)	0.1 M MMT (malate-MES-Tris) pH 4, 25% PEG 1500	0.1 M Na-acetate pH 4.6, 20 mM CaCl_2_, 30% MPD	0.1 M HEPES pH 7.5, 1 M Na-acetate, 50 mM CdSO_4_	0.1 M bis-Tris (pH 6.5), 2 M (NH_4_)_2_SO_4_	0.1 M bis-Tris-propane pH 6.5, 0.2 M (NH_4_)_2_SO_4_, 25% PEG 3350
Crystal handling	no cryosolution	no cryosolution (crystallization solution contained 30% MPD)	no cryosolution	cryosolution: 20 mM Tris pH 7.5, 0.1 M bis-Tris pH 6.5, 2.4 M (NH_4_)_2_SO_4_, 150 mM NaCl, 20% glycerol	no cryosolution
Resolution, Å	2.35	2.5–3.3	3.2	3.2	3.9
Protein conc. (mg/ml)	23	23 (seeding)	23	20.6	10.1
Temperature (°C)	20	20	20	20	20
Growth time (days)	8–12	1–4	3–6	8–14	7–10

Prior to crystallization, protein samples were additionally purified by SEC in 25 mM Tris pH 7.0–7.5 with 150 mM NaCl and with either 1 mM dithiothreitol (*) or 3 mM β-mercaptoethanol (**). PEG – polyethylene glycol; MPD – 2-Methyl-2,4-pentanediol; MES – 2-(N-morpholino)ethanesulfonic acid; Tris – tris(hydroxymethyl)aminomethane.

**Figure 3:**
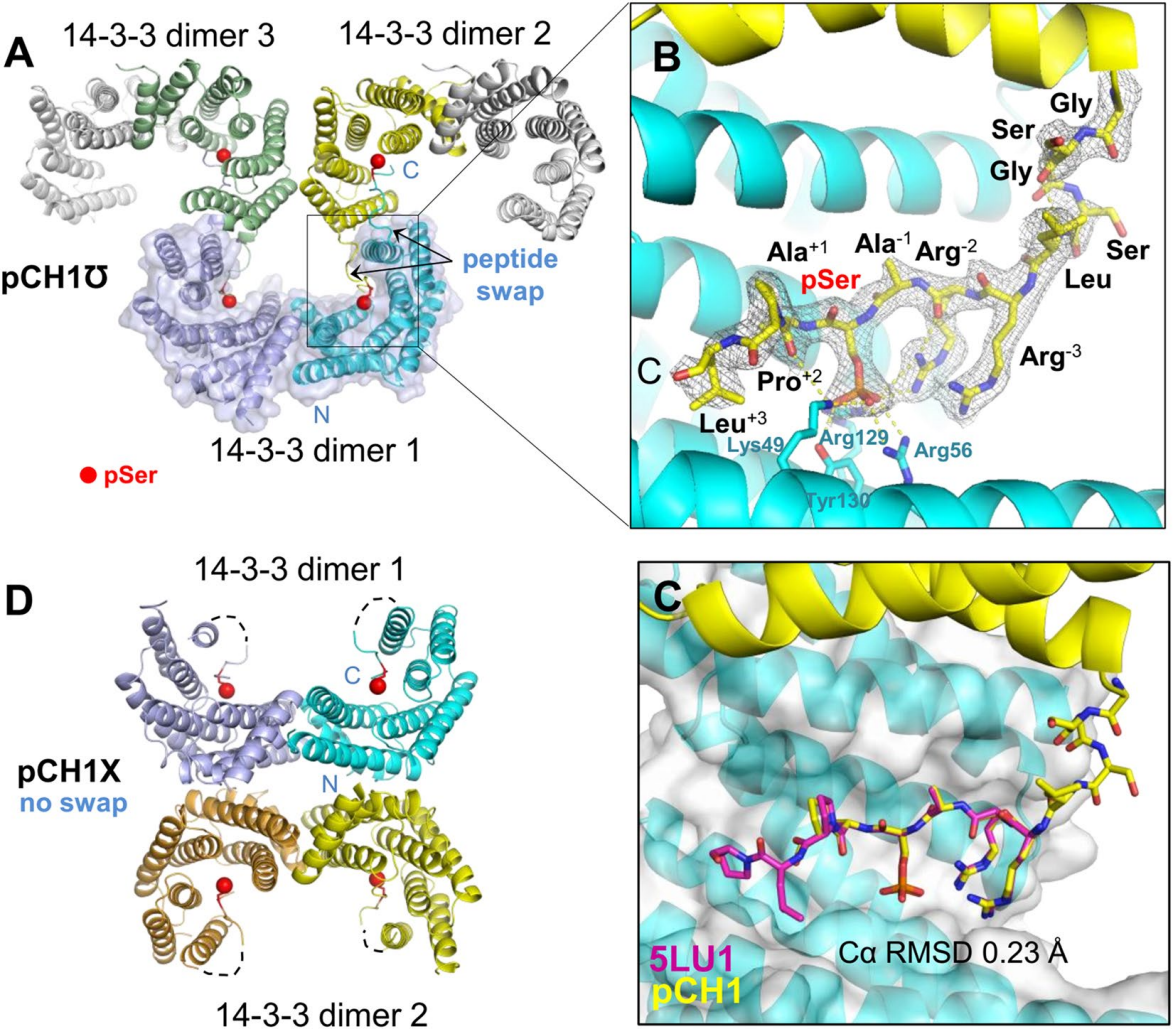
Crystal structures of the pCH1 chimeric protein. (**A**) – molecular packing in the pCH1℧ crystal form with the phosphopeptide (red sphere) swap between monomers of two 14-3-3 dimers. 14-3-3 subunits are shown as colored ribbons forming an inverted Ω shape; one physiological 14-3-3 dimer is highlighted by a semitransparent surface. (**B**) – magnified view showing the linker and the phosphopeptide with the corresponding 2F_o_-F_c_ electron density contoured at 1σ (residues are labeled, with numbers indicating positions with respect to pSer). (**C**) – Comparison of phosphopeptide conformations in the pCH1 (this work) and 5LU1 (synthetic HSPB6 phosphopeptide co-crystallized with 14-3-3σ^27^) structures. (**D**) – molecular packing in the pCH1X crystal form with no peptide swap (dashed lines correspond to unresolved parts of the linker).

Two crystal forms of the pCH1 chimera are remarkably distinct differing by the relative orientation and packing of 14-3-3 dimers in the crystal (Fig. 3). In one crystal form (pCH1℧, Table 1), the C-terminal lobes of each of the two subunits within a 14-3-3σ dimer are in contact with one C-terminal lobe in each of the two adjacent dimers (Fig. 3A). They form an interface along the length of the α-helix 9 of 14-3-3 stabilized by contacts between pairs of residues Tyr213/Tyr213′ and Gln221/Gln221′. As expected, the chimeric protein CH1 co-produced in bacteria with PKA was specifically phosphorylated at the authentic Ser residue (Ser16 of HSPB6^33^). In the structure, pairs of subunits belonging to two different 14-3-3 dimers are linked by a reciprocal *interdimer* phosphopeptide swap, in the course of which phosphopeptides, fused to the C-terminus of each subunit, cross-patch into the AG of the adjacent monomer. The electron density maps, calculated at 2.35 Å resolution (Fig. 3B and Table 2), allow unambiguous tracing of amino acids for a complete C terminus of the pCH1 chimera, including all residues of the linker with the exception of leucine at position +3 relative to pSer16. Lying just outside the primary 14-3-3 binding motif, RXXpSXP, this residue has no clear electron density suggesting its conformational variability. Notably, although being very short, the GSGS linker was long enough to allow phosphopeptide binding to the 14-3-3 monomer of an adjacent dimer.

**Table 2:** X-ray data collection and refinement statistics.

	pCH1℧	pCH1X	pCH2	pCH3
**Data collection**				
Space group	*P* 1 *2*_*1*_ 1	*P 2*_1_ 2_1_ 2_1_	*P 6*_4_ *2 2*	*P 4*_1_ 2_1_ 2
Cell dimensions: *a*, *b*, *c* (Å)	63.6, 140.6, 68.7	77.4, 97.8, 158.8	110.4, 110.4, 174.1	123.3, 123.3, 162.4
α, β, γ (°)	90, 114.8, 90	90, 90, 90	90, 90, 120	90, 90, 90
Resolution range (Å)*	**47–2.35** [47–6.6] (2.51–2.35)	**46.5–3.2** [46.5–6.9] (3.31–3.20)	**48–3.2** [48–9.4] (3.38–3.19)	**49–3.9** [49–11.4] (4.12–3.89)
Wavelength (Å)	0.9795	0.9795	0.9795	0.9282
*R*_merge_**	0.19 [0.07] (1.2)	0.45 [0.08] (3.1)	0.23 [0.03] (4.3)	0.30 [0.06] (2.9)
*R*_meas_	0.20 [0.08] (1.4)	0.49 [0.08] (3.4)	0.23 [0.03] (4.4)	0.30 [0.06] (3.1)
<*I*/σ>	6.5 (1.2)	4.4 (0.7)	14.3 (0.9)	6.8 (1.0)
*CC*_*1/2*_	0.99 (0.5)	0.99 (0.3)	1.00 (0.5)	1.00 (0.3)
Completeness (%)	95.5 (84.6)	99.8 (99.9)	99.6 (98.2)	99.6 (98.4)
Redundancy	3.9 (3.8)	6.5 (6.7)	23.0 (22.3)	12.8 (12.7)
**Refinement**				
No. of reflections: total	43838	20548	10910	12947
‘free’ set	1385	1016	977	1049
*R*_work_(%)	19.1	24.7	21.5	20.9
*R*_free_ (%)	24.0	27.9	26.7	24.8
No. of 2:2 complexes/asu	2	2	1	2
No. of non-H atoms: protein/ligands/solvent	8071/35/493	7327/17/22	3655/40/7	7246/38/1
R.m.s.d. bond lengths (Å)/angles (°)	0.010/1.0	0.010/1.0	0.010/1.1	0.010/1.1
Ramachandran favoured/outliers (%)	97.7/0.1	98.1/0.1	96.0/0.4	96/0.6
Molprobity score/Clash score	1.3/0.99	1.6/1.05	1.9/2.07	2.1/3.04
PDB code	**5OK9**	**5OKF**	**5OM0**	**5OMA**

^*^Statistics for the lowest and highest resolution shells are indicated in square brackets and parentheses, respectively.

^**^All statistics as defined in XSCALE^54^.

Importantly, irrespective of the interdimer peptide swap, the phosphopeptide orientation and conformation were identical to that of the synthetic HSPB6 peptide co-crystallized with the 14-3-3σ (PDB ID 5LU1 and 5LU2^27^), with the C_α_ r.m.s.d. of 0.23 Å for the residue segment RRApSAP (Fig. 3C), indicating highly specific binding and absence of any significant steric hindrance caused by peptide fusion. Consistent with available 14-3-3/peptide crystal structures, in the pCH1℧ structure reported here, the phosphate moiety of the peptide forms salt bridging interactions with the conserved 14-3-3σ residues Lys49, Arg56, and Arg129 and a hydrogen bond with Tyr130 (Fig. 3B). The main-chain atoms of alanines immediately adjacent to the phosphoserine make hydrogen bonding interactions with 14-3-3 residues Asn226 and Asn175, as observed in the 5LU1 structure^27^. This suggests that the proposed chimeric proteins can in principle be used to gain structural insights into the 14-3-3 interaction with any potential phosphopartners. Fusing phosphopeptides to the 14-3-3 protein imposes a 1:1 molar ratio for the two interacting moieties, eliminating the need to synthesize peptides and to optimize their concentration for achieving high occupancy while ensuring production of diffraction quality crystals.

The equivalence of protein-peptide interactions in fusion constructs was confirmed by the second crystal form of pCH1, where in the ASU we found two 14-3-3σ dimers located “back-to-back” (pCH1X, Fig. 3D and Table 2). In this case, the fused phosphopeptides appear to be bound back into the AG of the same monomer, resulting in fully occupied AGs. Notwithstanding the lower resolution of the corresponding crystal structure (3.2 Å), most of the phosphopeptide residues and several linker residues were resolved in electron density maps, yielding essentially the same structure of the bound phosphopeptide as for the pCH1℧ crystal form.

Crystal structures of the pCH1 chimera supported conclusions drawn from SEC data. Hydrodynamics calculations by HydroPro^43^ using the crystallographic pCH1 dimer (Fig. 3D) or dimer of dimers stabilized by reciprocal interdimer phosphopeptide links (Fig. 3A) resulted in *R*_H_ values 34.6 and 49.8 Å, respectively, in excellent agreement with the SEC-derived values of 3.4 nm and 4.9 nm (Fig. 2A).

### Crystal structures of pCH2 and pCH3 chimeras

Having found that the prototypic chimera pCH1 was structurally equivalent to 14-3-3 complex with synthetic phosphopeptide (Fig. 1D), but superior for complex preparation and crystallization, we created two further 14-3-3 chimeras, pCH2 and pCH3, containing structurally uncharacterized phosphopeptide binding partners in an attempt to obtain the first structural information for these complexes.

PKA-dependent phosphorylation and subsequent interaction of the transcription factor Gli, a central player in Hedgehog signaling^35^, and the steroidogenic acute regulatory protein StARD1^36,44^ with 14-3-3 proteins have recently been reported, however, no accurate structural information on these complexes was available. The proposed 14-3-3 binding phosphopeptide RRAS^640^DPAQA is conserved in all Gli proteins, i.e Gli1, Gli2 and Gli3. StARD1 has two phosphopeptide motifs, RRSS^57^LLGS and RRGS^195^TCVL that are expected to interact with 14-3-3. For chimeric protein construction, we have chosen the main phospho-site of Gli for pCH2 and the first potential 14-3-3 binding motif of StARD1 (RRSS^57^LLGS) for pCH3. A similar phosphopeptide motif to StARD1 is found at Ser87 position of human Bcl-2-like protein 11 (BimEL11). CH2 and CH3 proteins were co-expressed with PKA and purified in a fully soluble form using the same protocol as for pCH1 (Fig. 1), indicating that the high inherent solubility of 14-3-3 is not compromised by peptide addition at its C terminus. Both proteins readily crystallized in various conditions producing diffraction-quality crystals of different morphologies straight out of commercial screens (see Table 1).

**Figure 4:**
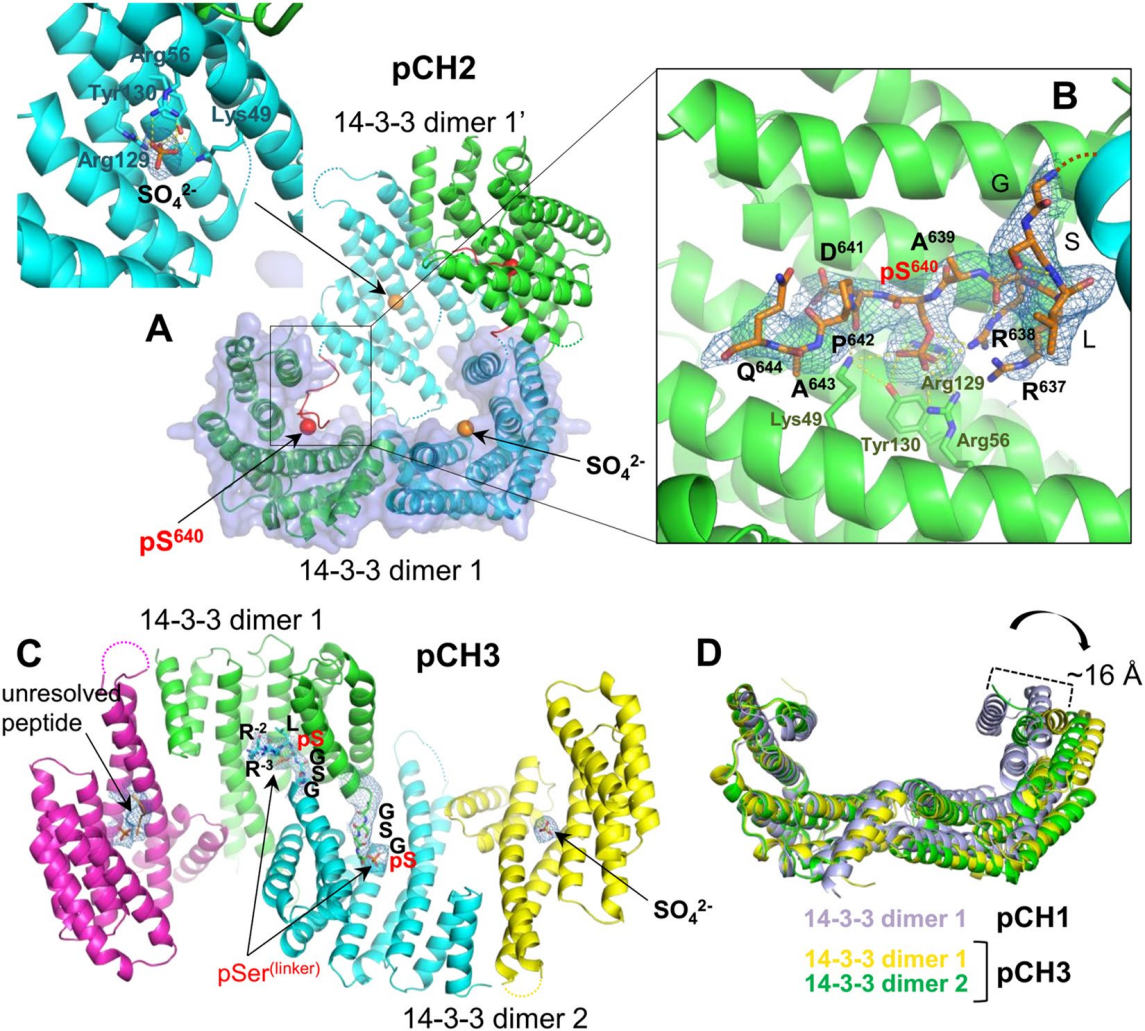
Crystal structures of pCH2 and pCH3 chimeric proteins. (**A**) – crystal packing of two adjacent dimers of the 14-3-3σ fusion with Gli phosphopeptide (pCH2), showing phosphopeptide exchange between the two dimers. A close-up view at the sulfate anion (orange) bound in the peptide-free AG of the phosphopeptide donor subunit (inset). (**B**) – Close-up view at the bound Gli phosphopeptide with 2F_o_-F_c_ maps contoured at 1σ and with indicated key residues. (**C**) – packing of two dimers of the 14-3-3σ fusion with StARD1/BimEL phosphopeptide (pCH3 chimeric protein). The phosphorylated linker GSGpSLRR, the sulfate anion and the unresolved phosphopeptide (indicated by labeled arrows) are shown with corresponding 2F_o_-F_c_ electron density maps contoured at 1σ. (**D**) – overlay of 14-3-3 dimers obtained by superposition of a single subunit, demonstrating conformational differences between the fully closed and open states, with up to ~16 Å positional differences.

The pCH2 crystal structure was determined at 3.2 Å resolution (Table 2). There is one 14-3-3 dimer per asymmetric unit; forming a closed cyclic structure with its symmetry mate where a single subunit of each dimer contributes its phosphopeptide to the binding site of the closest subunit of an adjacent dimer (Fig. 4A) having its own binding site occupied by a sulfate ion (sulfate was present in crystallization condition). In the inter-dimer complex, the central Ser640 is bound by the same canonical residues of AG as in pCH1 (Fig. 3B) and other 14-3-3/phosphopeptide complexes. Interestingly, this binding mode leaves the two opposite AGs unoccupied by the two remaining phosphopeptides which are unresolved in electron density maps. Instead, these ‘unbound’ AGs appear to be occupied by sulfate anions present in crystallization media at high concentration (Fig. 4A and Table 1). One can speculate that the very high sulfate concentration (2M) may have forced the phosphopeptides to partially dissociate from the AG facilitating this distinct crystal packing, in line with the inhibitory effect of phosphate and sulfate ions on 14-3-3/phosphotarget interactions observed *in vitro* ^24^. The quality of electron density maps allowed unequivocal tracing of all linker residues of the Gli-derived phosphopeptide, except for the C-terminal alanine (Fig. 4B). Interestingly, two closely packed phosphopeptide-binding 14-3-3 subunits belonging to two different dimers, form Cys38-Cys38 disulfide bridge. However, this interaction is distant to AGs of each subunit and does not appear to interfere with 14-3-3/phosphopeptide interactions. This example demonstrates that the 14-3-3/phosphopeptide fusions are useful for producing accurate structural information for 14-3-3 complexes with partners for which little or no information on binding affinity is available.

Unexpectedly, structural data obtained for the pCH3 chimera were significantly different. Most crystals produced poor diffraction, with the best 3.9 Å resolution data set collected from a crystal with two trans-interacting dimers in the ASU (Fig. 4C and Table 2). The electron density maps of two neighboring subunits belonging to two different 14-3-3 dimers were clear enough to trace the C-terminal portion and the following peptide and to conclude that it binds in the AG of the adjacent subunit. Although the distance between the C-terminal tryptophan of the 14-3-3 core and the positively charged residues typically coordinating the phosphoserine, was too short to allow canonical phosphopeptide binding, binding of a phosphorylated residue was still observed (Fig. 4C). Surprisingly, in both cross-patching peptides, we found that it was the second Ser residue of the linker (-GSGpS-) that was phosphorylated and bound in the adjacent subunits’ AG (Fig. 4C). In one of the chains, we could trace three residues beyond this pSer, LRR, indicating that the bound peptide had a completely reversed orientation to those observed for pCH1, pCH2 and other known 14-3-3/phosphopeptide structures. This clearly exemplified the extremely rare case when a peptide phosphorylated by PKA at a non-canonical position (N-..pSXXR..-C instead of N-..RXXpS..-C) was then erroneously accommodated in the AG of 14-3-3 but in the non-standard, opposite orientation. This could be an artifact caused by a relatively high level of PKA co-expression, reducing phosphorylation specificity, although under the same conditions this did not happen to pCH1 and pCH2, where the same linker remained unphosphorylated. We note that an atypical reversed orientation of the peptide fragment in the AG of 14-3-3ζ has been previously reported for the Exoenzyme S (PDB ID 2O02). We do not know the phosphorylation status of the target and neighboring serine residues (RRSSLLGSR, underlined), however, to exclude atypical phosphorylation, serine residues in the GSGS linker could be avoided. Although it was not possible to observe the canonically bound conformation of the StARD1/BimEL phosphopeptide, this remarkable crystal structure illustrates the promiscuity of the 14-3-3 groove, exemplifying multiple exceptions to the established canonical binding rules based on motifs I-III^13,15^. This example further demonstrates the value of 14-3-3 chimeras for structural studies of weakly binding phosphopeptides which differ significantly from the canonical higher affinity motifs.

Interestingly, in the pCH3 structure, both 14-3-3 dimers adopted a significantly more open overall conformation than in the pCH1 or pCH2 cases (Fig. 4D). Superposition of one of the 14-3-3 dimer monomers showed that the extreme position of the C-terminal α-helix of the second monomer differed by up to 16 Å (dimer 1 of pCH3 had an intermediate position), reminiscent of the quite unique structure of the apo-14-3-3β isoform with one subunit in a closed and one in an open conformation^6^. Surprisingly, in the pCH3 structure, the open conformation of the 14-3-3σ subunit did not prevent binding of either the sulfate ion or a phosphorylated peptide (Fig. 4C, yellow). Accurate structural data on different 14-3-3 conformations support earlier predictions by molecular dynamics^41^.

## Discussion

Progress in translating the wealth of available knowledge on 14-3-3 proteins into novel therapeutic approaches is often limited by the lack of accurate structural information on specific complexes of these hub proteins with interacting partners. We attempted to resolve this problem by designing chimeric proteins where the phosphopeptide binding partner is fused to a 14-3-3 core. We probed whether such chimeric proteins are soluble and whether they are suitable for structural studies by protein crystallography. Our data demonstrate that chimeras can be used for setting up a streamlined and highly efficient protein crystallization pipeline for rapid generation of structural information for previously uncharacterized 14-3-3 target phosphopeptides, opening up new perspectives in 14-3-3 research.

One of the advantages of using the 14-3-3/phosphopeptide chimeras is that they are easy to design and produce in a soluble form in *E. coli*, as solubility is conferred by the highly soluble 14-3-3 protein and phosphorylation is achieved by co-expression with a protein kinase. PKA, used in this work for co-expression, may be substituted by the cognate kinase known to phosphorylate the target 14-3-3 binding site, provided that it is sub-cloned into a compatible expression vector and is soluble in *E. coli*. Alternatively, *in vitro* phosphorylation of purified 14-3-3 chimeras (see Fig. 1A, inset) by commercially available protein kinase(s) is also an option.

The established purification protocol is affordable and straightforward leading to production of large amounts (>10 mg per liter of culture) of highly pure (>98%) and monodispersed protein suitable for subsequent crystallization experiments. The presence of the core 14-3-3 construct optimized for crystallization facilitates production of diffraction quality crystals, straight from commercial screens. Additionally, chimera/peptide libraries can be easily created, since the peptide-encoding DNA can be readily inserted into the chimera expression system using synthetic oligonucleotides and current molecular biology protocols. These advantages make the approach adaptable for high-throughput studies, such as screening for novel 14-3-3 protein interacting partners, validation of newly identified protein-protein interactions involving 14-3-3, and screening for small molecule modulators of the established 14-3-3/phosphotarget complexes.

The inevitable substantial advantage of the proposed chimeric 14-3-3/phosphopeptide constructs is that the covalent tethering ensures 1:1 stoichiometry. In contrast, traditionally utilized synthetic peptides can be labile and/or of limited solubility^27^ and hence crystallization may be inhibited by a large excess of a peptide while too little peptide may result in partial occupancy of the AG of 14-3-3. This is especially important for weak binding peptides where the apparent decrease in dissociation constant, due to the significant increase in local phosphopeptide concentration when fused to 14-3-3, can assist in obtaining a high binding occupancy of the partner AG site. Fusion of such peptides to 14-3-3 with the help of a carefully designed linker presents a unique opportunity to obtain corresponding structural information about their conformation in the AG of 14-3-3.

The optimal linker length, often an Achilles’ heel in fusion proteins, was based on the crystal structure of the exotic 14-3-3 protein Cp14b, bound to its own phosphorylated C terminus (Fig. 1A). The approach led to the successful structure determination for several 14-3-3/phosphopeptide complexes (Figs 3 and 4). Although the structure of a 14-3-3ζ chimera with a pseudophosphorylated peptide (S → E substitution) from the tumour suppressor LKB1 was reported recently (PDB ID 4ZDR), the mutation or non-optimal (longer) linker resulted in a surprising and most likely unspecific binding of a peptide, manifested by different binding to each of the two subunits of the 14-3-3 dimer present in the asymmetric unit^45,46^. Surprisingly, the primary binding site in the 4ZDR structure is occupied by a sulfate anion, suggesting that the S → E mutation is a poor mimic of phosphorylation. Thus, the observed peptide conformation^45^ cannot be considered as genuine. In contrast, phosphopeptide conformations observed for the pCH1 chimera structures reported here were validated by direct comparison with the structure of 14-3-3σ complex with synthetic HSPB6 phosphopeptide (PDB ID 5LU1). The comparison showed that the two different approaches provided almost identical structural information (Fig. 3C), with the C_α_ r.m.s.d. of 0.23 Å for bound peptides.

Interestingly, phosphopeptide binding within the pCH1 chimera resulted in protein compaction and a significant increase in thermal stability, as evidenced by analytical SEC and fluorescence spectroscopy (Fig. 2), in line with partial stabilization of 14-3-3 by phosphate and phosphopeptides observed earlier^47^. Such observations can be used to probe the folding and stability of other 14-3-3 chimeras prior to crystallization and may be also useful for screening for small molecule inhibitors of 14-3-3/partner interaction.

The approach based on the 14-3-3 chimera scaffold, that we introduced here (Fig. 1), can facilitate structural studies of more complicated 14-3-3 complexes, particularly those where binding partners have a single 14-3-3-binding site located at their N terminus. For example, *ternary* complexes involving 14-3-3 scaffolds, long hypothesized but poorly evidenced so far, could now be studied with increased confidence. One possibility would be to use heterodimeric chimeras created through fusion of two different phosphopartner peptides to different 14-3-3 isoforms known to preferentially heterodimerize^4–6^. For other assemblies, where binding of a protein or domain to 14-3-3 is only possible after phosphopeptide binding, 14-3-3 chimeras could be used as preformed binding partners. Examples include the ternary 14-3-3 complex, GF14c/OsfD1/Hd3a, that regulates flowering in plants^48^ or the mammalian 14-3-3/HSPB6 regulatory complex, where binding of the alpha-crystallin domain of HSPB6 most likely takes place after 14-3-3/phosphopeptide binding in the AG^27^. The modular principle of the chimeras described in this study could also be adaptable to study phosphoserine/threonine binding proteins more generally^49^.

In summary, we present a simple but powerful approach for rapid production of accurate X-ray structures for 14-3-3 proteins bound to partner phosphopeptides. We tested this approach by determining structures of 14-3-3/phosphopeptide complexes and present structural information for novel phosphopeptide complexes of 14-3-3. The data provided by these and future structures, produced using this approach, will deepen our understanding of the factors dictating phosphopeptide target selection by 14-3-3 proteins, informing the potential development of new therapies based on targeting specific protein interactions.

## Methods

### Cloning, expression and purification of 14-3-3 chimeras

Cloning, overexpression and purification of the monomeric mutant form of human 14-3-3ζ (14-3-3ζ_m_: ^12^LAE^14^ → ^12^QQR^14^ ^14^) and the untagged C-terminally truncated human 14-3-3σ (14-3-3σ∆C: residues 1-231) were described previously^27,39,40^. To facilitate crystallization of the protein, we followed the so-called surface-entropy reduction (SER) approach^50^ and cloned 14-3-3σ∆C mutant Clu3 with ^75^EEK^77^ → ^75^AAA^77^ amino acid replacements into a modified pET28 vector containing a 3C-cleavable N-terminal hexahistidine tag^27^. cDNA of the 14-3-3 chimera with the HSPB6 peptide RRAS^16^APL (CH1) was obtained in one PCR step using the pET28-his-3C_14-3-3σ∆C-Clu3 construct as a template by high-fidelity *Pfu* polymerase using T7-forward 5′-GACTCACTATAGGGAGACC-3′ and an excess of Clu3-B6p reverse primer 5′-ATATCTCGAG*TCA*CAACGGGGCGCTAGCGCGGCGCAGGGATCCCGATCCCGTCCACAGTGTCAG-3′ introducing the HSPB6 peptide and linker (GSGS) sequences, and *XhoI* site. cDNA of the 14-3-3 chimeras with the Gli (CH2) or StARD1/BimEL (CH3) peptides were obtained on the basis of CH1 by the same procedure as for CH1 but using 5′-ATATCTCGAGTCATGCTTGAGCAGGATCACTAGCGCGGCGCAG-3′ or 5′-ATATCTCGAGTCAACGAGATCCCAGCAGGCTGCTGCGGCGCAGGGATC-3′ reverse primers, respectively, introducing the peptide and linker sequences and *XhoI* site. cDNA of CH1-CH3 was subsequently cloned into pET28-his-3C vector using *NdeI* and *XhoI* sites for restriction endonucleases and T4 DNA-ligase (SibEnzyme; www.sibenzyme.com). Correctness of all constructs was verified by DNA sequencing in Evrogen (www.evrogen.com). The plasmid encoding the CH1 chimera created in this study is deposited with Addgene (www.addgene.org) under the accession number 100093. Other plasmids are available from the corresponding author on reasonable request.

All phosphorylated chimeras (pCH1-pCH3) were obtained according to the identical scheme. Corresponding constructions in pET28-his-3C vector (kanamycin resistance) were used for co-transformation and co-expression in *E. coli* with a His-tagged catalytically active subunit of mouse PKA cloned in pACYC vector (chloramphenicol resistance)^27^ under selection on both antibiotics. CH1 was also obtained in an unphosphorylated state, i.e. via expression in the absence of PKA. Protein overexpression in 1 L of Luria-Bertani media was induced at OD_600_ of 0.6 by addition of isopropyl-β-D-thiogalactoside to a final concentration of 0.5 mM for 20 h at 30 °C.

Purification was performed using subtractive immobilized metal-affinity chromatography (IMAC) and gel-filtration essentially as described^27^. Between IMAC1 and IMAC2 steps (loading/washing buffer (A): 20 mM Tris pH 8.0, 300 mM NaCl, 10 mM imidazole; elution buffer (B): buffer A with additional 500 mM imidazole) the chimeras were dialyzed to remove imidazole and simultaneously cleaved with 3C protease^27,51^ (1:1000 weight 3C: chimera ratio estimated by absorbance at 280 nm) resulting in target proteins with three extra residues GPH- at their N terminus. The final polishing size-exclusion chromatography step was immediately followed by screening for crystallization conditions or *in vitro* characterization. The amount of protein obtained from 1L of bacterial culture was usually enough to setup exhaustive initial screening and obtain diffraction quality crystals without further optimization. All final protein samples were homogenous according to a Coomassie-stained SDS-PAGE. Protein concentration was determined spectrophotometrically at 280 nm.

### Native gel-electrophoresis

Phosphorylation and dephosphorylation of CH1 *in vitro* were performed as described elsewhere^37^. The results were analyzed by 15% polyacrylamide gel-electrophoresis at pH 8.6 under non-denaturing conditions.

### Analytical size-exclusion chromatography

The oligomeric status and hydrodynamic properties of 14-3-3ζ_m_ and CH1 or pCH1 were assessed and compared using analytical SEC, as described previously^52^. 100 µL protein samples were pre-incubated for 30 min at room temperature and then loaded on a Superdex 200 Increase 10/300 column (GE Healthcare) equilibrated with a 20 mM Tris-HCl buffer, pH 7.6, containing 150 mM NaCl, 0.1 mM EDTA, and 3 mM β-mercaptoethanol (ME), at a flow rate of 1.2 mL/min, while monitoring absorbance at 280 nm. The column was calibrated with protein standards with known hydrodynamic radii that were used to determine average radii *R*_H_ of the species under study^52,53^. Profiles were built using *Origin 9.0 Pro* software.

### Fluorescence spectroscopy

To get insight into thermal stability of proteins, we monitored changes in the intensity of intrinsic tryptophan fluorescence at 320 (*I*_320_) and 365 (*I*_365_) nm upon excitation at 297 nm (slits width 5 nm) during heating of the samples (1–5 µM protein concentration on a 20 mM Hepes buffer, pH 7.1, 150 mM NaCl, 0.1 mM EDTA, 2 mM ME) from 10 to 80 °C at a constant rate of 1 °C/min in a temperature-controlled multicell holder of a Cary Eclipse fluorescence spectrophotometer (Varian Inc.). Before the experiment, the samples were equilibrated for 10 min at the initial temperature (10 °C). The ratio of *I*_320_(T)/*I*_365_(T) normalized from 0 to 100% represented the dependence of completeness of thermal transition, of an unfolded fraction, on temperature and was used to estimate half-transition temperatures^42^. When possible, the single wavelength was used to build analogous transition curves^53^. Graphs were built using *Origin 9.0 Pro* software.

### Crystallization and X-ray data collection

The 14-3-3 chimeras were subjected to crystallization trials immediately after purification using commercial screens PACT, Procomplex (Qiagen), Index, Crystal Screen (Hampton Research) and JCSG + (Molecular Dimensions). Sitting drops containing 200 nl protein at 10–23 mg/ml concentration (See Table 1) and 100–200 nl precipitant solution were set up in 96-well plates using the Mosquito robot (TTL Labtec). Crystals were difficult to optimize, however, in some cases random matrix microseeding appeared helpful (Table 1). Crystallization plates were incubated at 20 °C and monitored using a Rigaku plate imager equipped with a Vis/UV-scanning and detection system.

X-ray diffraction data (Table 2) on small crystals, grown directly in 96-well plates, were collected at 100 K at beamlines I02 and I04 of Diamond Light Source (UK) using Dectris PILATUS 6MF detectors. Crystals were mounted in nylon loops and quickly cooled in liquid nitrogen, predominantly without addition of a cryoprotectant (See Table 1 for details).

### Crystal structure solution and refinement

Diffraction data were integrated and scaled using *XDS/Xscale* ^54^ and *xdsme* ^55^. Phasing of the pCH1-pCH3 was accomplished by molecular replacement with *MolRep* ^56^ using the dimer of the 14-3-3σ Clu3 mutant from the PDB ID 5LU1 as a search model. Initial phasing attempts in the case of the pCH3 using the 14-3-3σ dimer failed. However, it was possible to solve the structure using the 14-3-3σ monomer as a search model, with molecular replacement placing three out of four subunits in the ASU, and with the fourth subunit that had a substantially different (more open) overall conformation recovered in *Coot* ^57^ by manual placing of α-helices into electron density maps calculated with phases based on the three initial 14-3-3 monomers. The missing C-terminal segments containing fused phosphopeptides and sulfate anions were manually built into difference electron density maps. Automated refinement in *Buster* 2.10.3^58^ initially included a rigid-body refinement of all chains and then an all-atom and individual B-factor restrained refinement. Statistics of final refined models are in Table 2. The relatively high R-factors in the case of the pCH1X structure can be caused by a pronounced translational NCS detected for this crystal form, which significantly complicated the refinement. In this case, *Zanuda* ^59^ was used to validate the *P* 2_1_ 2_1_ 2_1_ space group.

All figures depicting the structure were prepared using Pymol 1.6.9 (Schrödinger). Atomic coordinates and structure factors have been deposited with the PDB under accession codes indicated in Table 2. All other data generated during the current study are included in this article.
